# Inguinal draining-lymph node in ^18^F-FDG PET/CT images could be a new indicator for the diagnosis of fracture-related infection in the lower extremities

**DOI:** 10.3389/fimmu.2023.1206682

**Published:** 2023-10-05

**Authors:** Yanbing Wang, Zhenkui Sun, Xiao Liang, Chentian Shen

**Affiliations:** ^1^ Department of Nuclear Medicine, Rizhao People’s Hospital, Rizhao, Shandong, China; ^2^ Department of Nuclear Medicine, Shanghai Sixth People’s Hospital Affiliated to Shanghai Jiao Tong University School of Medicine, Shanghai, China; ^3^ Bone Nonunion and Bone Infection Multidisciplinary Team (MDT), Shanghai Sixth People’s Hospital Affiliated to Shanghai Jiao Tong University School of Medicine, Shanghai, China; ^4^ Department of Radiology, Rizhao People’s Hospital, Rizhao, Shandong, China

**Keywords:** fracture-related infection, 18 F-FDG PET/CT, lymph node, standardized uptake value, lower extremity

## Abstract

**Purpose:**

The imaging diagnosis of fracture-related infection is often challenging. The aim of this study was to evaluate the value of ^18^F-FDG PET/CT for the diagnosis of fracture-related infection (FRI) with internal fixation after orthopedic surgery in lower extremities.

**Methods:**

A total of 254 consecutive patients who underwent ^18^F-FDG PET/CT scans with suspected FRI with internal fixation in lower extremities were retrospectively investigated ^18^F-FDG PET/CT images were semiquantitatively evaluated with multiple metabolic parameters. Additionally, morphological information of the inguinal draining lymph nodes (DLN) with the highest SUV value was also collected and analyzed.

**Results:**

Patients were divided into two groups according to final diagnosis: the infected (N=197) and the non-infected group (N=57). The differences in the inguinal DLN-related parameters, including the long diameter, short diameter, maximum cross-sectional area, maximum standardized uptake value (SUVmax), metabolic volume (MV) 60%, MV70%, MV80%, total lesional glycolysis (TLG) 60%, TLG70%, TLG80%, and the infection suspected area related parameters, including SUVmax, MV25%, MV30%, MV35%, MV40%, MV50%, and TLG70%, between the two groups were statistically significant. We then compared the highest area under the curves (AUCs) among the morphological parameters of DLN, metabolic parameters of DLN, and metabolic parameters of the suspected infection area. The result demonstrated that SUVmax of the inguinal DLN showed the best diagnostic performance with an AUC of 0.939 (*P*<0.05).

**Conclusion:**

Semiquantitative analysis (especially SUVmax) of the inguinal DLN in ^18^F-FDG PET/CT images could be a promising method for the diagnosis of suspected FRI with internal fixation after orthopedic surgery in lower extremities.

## Introduction

Fracture-related infection (FRI) is one of the most prevalent and challenging complications followed by trauma surgery, especially in patients receiving orthopedic implants (1–4). The most common microorganisms causing implant-associated infections are Staphylococcus aureus and Coagulase-negative staphylococci (5). Infection is among the main causes of persistent non-union fractures and thus osteosynthesis failure, which requires invasive treatment and is a major burden for healthcare systems (6). What is more, the growing amount of placed fracture-related implants and patients with inducing conditions (e.g., diabetes mellitus and peripheral vascular diseases) may increase the incidence of FRI (7, 8).

The timely identification of infection after orthopedic surgery is essential for effective treatment (9). A consensus on how to diagnose FRI was made by the AO Foundation and European Bone and Joint Infection Society (EBJIS) recently (10), which compromised a set of confirmatory criteria (infection definitely present: for instance, fistula, sinus, purulent drainage, and the presence of pus) and suggestive criteria (infection possibly present: for instance, related clinical manifestations and radiological imaging). The evaluation of suspected FRI can be characterized by a multimodality workup containing physical and imaging examination and laboratory parameters (i.e., an elevated erythrocyte sedimentation rate and C-reactive protein). However, FRI does not always announce itself with evident symptoms. Therefore, a definite diagnosis of FRI could sometimes be quite challenging (11).

Conventional imaging methods such as plain X-rays and computed tomography (CT) can help little for the diagnosis of FRI due to nonspecific imaging features (12). Magnetic resonance imaging (MRI) can distinguish osteomyelitis with high certainty (13). However, the diagnostic value of MRI is weakened for FRI because sterile inflammation and reparative fibrovascular scar tissue usually show similar signal intensities such as reactive marrow edema (14–16). Furthermore, MRI images can be hampered by the presence of internal fixation material (17). Considering its pathophysiological processes, multiple nuclear imaging modalities such as three-phase bone scintigraphy (BS), leukocyte scintigraphy (namely, white blood cell [WBC] scintigraphy), and ^18^F-fluorodeoxyglucose (^18^F-FDG) positron emission tomography (PET)/computed tomography (CT) have been investigated to evaluate the diagnostic efficacy of orthopedic surgery related infections including periprosthetic joint infection and FRI (18).

In a previously published meta-analysis, ^18^F-FDG PET/CT scans have presented satisfactory accuracy for the diagnosis of osteomyelitis compared with bone scintigraphy and leucocyte scintigraphy (19). Institutions using ^18^F-FDG PET/CT to evaluate suspected FRI are steadily growing worldwide, and this nuclear medicine imaging modality showed encouraging diagnostic accuracy based on qualitative assessments (20–24). Uptake of ^18^F-FDG reflects cellular glucose metabolism and can be semiquantitatively expressed as SUV, MV, TLG, etc. Increased glucose consumption induces increased ^18^F-FDG-uptake in the areas with enhanced metabolic activity of the tissue involved or the invasion of inflammatory cells (25–27). In addition, studies also evaluated the value of semiquantitative measurements in ^18^F-FDG PET/CT for the diagnosis of suspected infections for non-union fractures, and they achieved satisfactory diagnostic accuracy (28, 29). Results from previous studies indicated that ^18^F-FDG PET/CT could play an essential role in the diagnosis of FRI.

In clinical practice, increased uptake of ^18^F-FDG in inguinal lymph nodes can be frequently found during PET/CT scans in patients with suspected FRI in lower extremities. Enlargement of draining lymph nodes (DLNs) is related to infection, sterile inflammation, autoimmune disorder, cancer, and other diseases where the immune system is involved. The immune response in the DLNs can be etiology and pathogenesis specific (30–34). Thus, we supposed that the ^18^F-FDG uptake pattern in the inguinal DLNs could be different in fracture-related infections and aseptic inflammation in the lower extremities. To the best of our knowledge, the diagnostics value of DLNs in ^18^F-FDG PET/CT images for FRI has not been reported. The aim of our study, therefore, was to further evaluate semiquantitative measurements of ^18^F-FDG PET/CT for the diagnosis of suspected FRI with internal fixation in lower extremities and to focus not only on the suspected infection area but also the DLNs in the inguinal region.

## Methods

### Patients

We retrospectively enrolled 254 consecutive patients of suspected FRI with internal fixation in lower extremities who had undergone ^18^F-FDG PET/CT scan in Shanghai Sixth People’s Hospital between Dec 2015 and July 2021. The surgery following was performed within 1 month. The time interval between the latest fracture surgery and the PET/CT scan was more than 3 months in all patients. The final diagnosis of FRI was confirmed according to the consensus (10) made by the AO Foundation and EBJIS. Of the 254 patients, 197 were diagnosed as having an infection (89 with positive bacterial culture; 108 with negative bacterial culture), and 57 were diagnosed as having aseptic inflammation.

### ^18^F-FDG PET/CT acquisition protocol

Before ^18^F-FDG PET/CT scans, patients were required to fast for at least 6 hours with a maximum blood glucose level of 11 mmol/L. Scans were performed 40-60mins after intravenous administration of 3.7MBq/Kg dose of ^18^F- FDG by using an integrated PET/CT scanner (Discovery VCT, GE, USA). PET/CT scans of the lower extremities were performed from the anterior superior spine to toes with the following settings: CT scan, 120V and 80mA, 64 slices, 3.75mm slide thickness; PET scan, 3D model, 2.5 min/bed. PET images were then reconstructed iteratively by using ordered subset expectation maximization (OSEM), and attenuation correction was applied according to CT for all scans.

### Image analysis

^18^F-FDG PET/CT images were interpreted semiquantitatively by two experienced senior nuclear medicine physicians. For the semiquantitative analysis, the volume of interest (VOI) was set in the target area with suspected infection and the corresponding inguinal draining lymph node with the highest SUVmax value. The semiquantitative parameters, including the maximum standardized uptake value (SUVmax), metabolic volume (MV)25%, MV30%, MV35%, MV40%, MV50%, MV60%, MV70% of suspected infection area, and SUVmax, MV60%, MV70%, MV80% of the inguinal lymph node within the VOIs, were detected. The corresponding total lesional glycolysis (TLG) value (TLG25%, TLG30%, TLG35%, TLG40%, TLG50%, TLG60%, and TLG70%) was calculated according to the following formula: TLG = SUVmean × MV (with corresponding SUV threshold). In addition to the above metabolic parameters, morphological information in CT images, including long diameter, short diameter, and maximum cross-sectional area of the inguinal DLN (with the highest SUVmax value), was also collected and analyzed.

### Statistical analysis

The statistical analysis was performed using IBM SPSS version 26.0 and MedCalc version 19.2.6. Comparisons were assessed using the Student’s *T* test (parametric data) or the Mann–Whitney *U* test (non-parametric data). Data were expressed as mean ± standard deviation (SD). Receiver operating characteristic (ROC) curves for parameters were plotted, and the corresponding area under the curve (AUC) values were computed. The diagnostic sensitivity and specificity of all parameters were calculated. Differences of AUCs among parameters were compared by MedCalc. A *P* value < 0.05 was taken as the threshold of statistical significance.

## Results

### Patient cohort

The mean age of the patients was 47 ± 15 years (range 12-81). Of the 254 patients, 61 (23.9%) were women and 193 (75.7%) men. Patients were divided into two groups according to final diagnosis: the infected (N=197) and the non-infected group (N=57). A total of 86 patients were infected by a single pathogen (Staphylococcus aureus, N=41; Staphylococcus epidermidis, N=2; Enterococcus faecalis, N=8, Enterobacter cloacae, N= 7; Pseudomonas aeruginosa, N = 11; Klebsiella pneumoniae, N=2; the others with rare pathogens), and 3 patients were infected by mixed bacteria (Morgan bacteria and Staphylococcus aureus; Pasteurella aerogenes, Pseudomonas aeruginosa and Staphylococcus aureus; Staphylococcus aureus and Staphylococcus epidermidis, respectively). Patient characteristics are summarized in **Table 1**.

**Table 1:** Clinical characteristics for patients in infected and non-infected groups.

Characteristics	Total N=254	Infected group N=197	Non-infected group N=57
Age(years)			
(mean ± SD)	47 ± 15	46± 16	51± 14
Gender			
Male	193	155	38
Female	61	42	19
Location of non-union			
Femur	90	52	38
Tibia	105	95	10
Femur and tibia	1	1	0
Fibula	4	4	0
Tibia and Fibula	46	37	9
Patella	2	2	0
Calcaneus	6	6	0

### Diagnostic performance of different parameters in ^18^F-FDG PET/CT images

**Figure 1:**
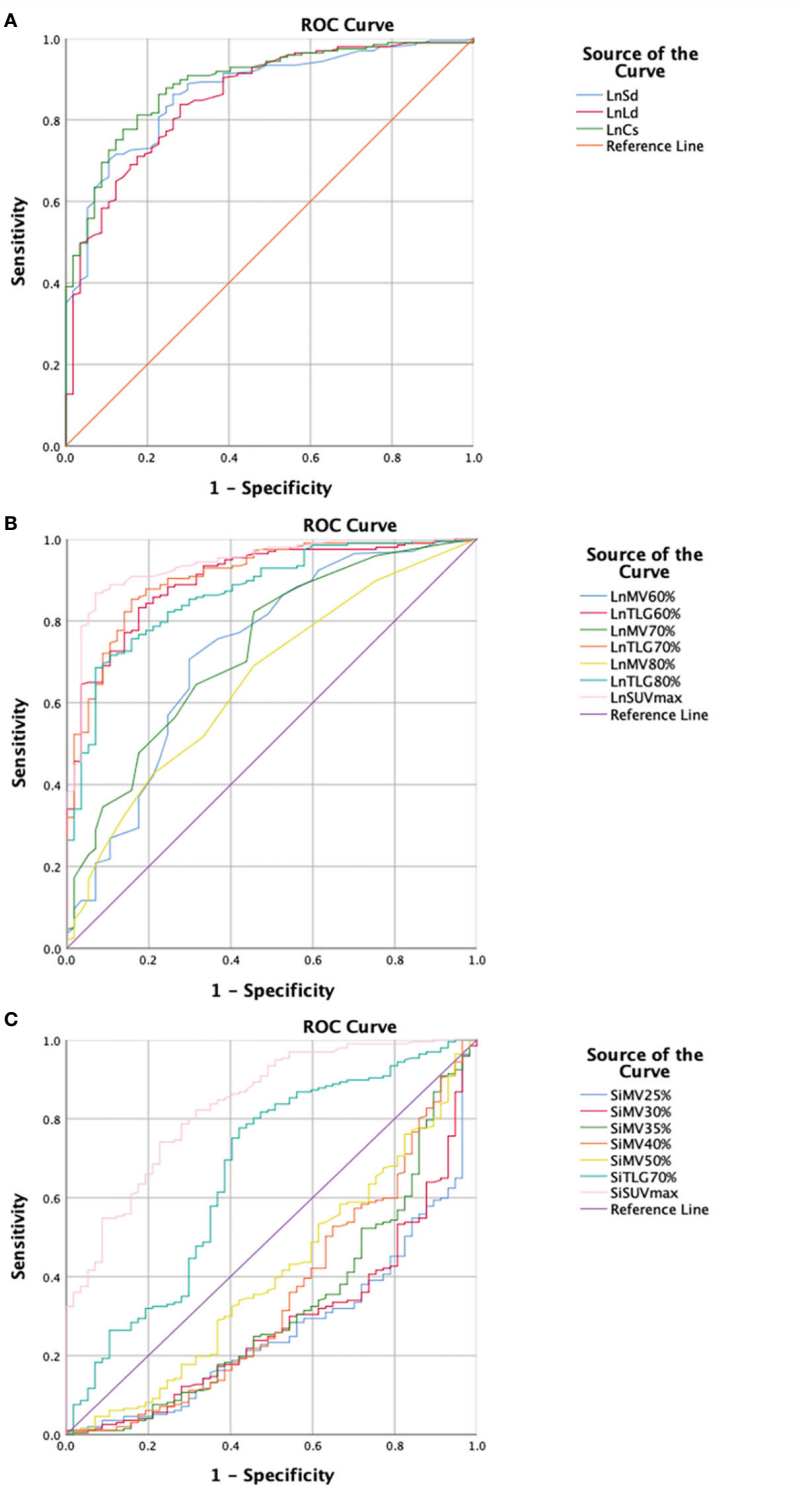
Receiver operating characteristics (ROC) curves of the parameters analyzed in this study regarding morphological and metabolic parameters of the inguinal draining lymph node (DLN) as well as metabolic parameters of the infection suspected area. **(A)** Morphological parameter of the inguinal DLN. The maximum cross-sectional area of DLN shows the highest area under curve (AUC) (0.889). The sensitivity and specificity are 81.2% and 82.5%, respectively, with a cut-off value of 117.27mm^2^. **(B)** Metabolic parameters of the inguinal DLN. SUVmax of DLN shows the highest AUC (0.939). The sensitivity and specificity are 86.8% and 93.0%, respectively, with a cut-off value of 1.55. **(C)** Metabolic parameters of the infection suspected area. SUVmax of suspected infection area shows the highest AUC (0.836). The sensitivity and specificity are 74.0% and 77.2%, respectively, with a cut-off value of 4.53. DLN, draining lymph node; Ln, lymph node; Sd, short diameter; Ld, long diameter; Cs, cross-sectional area; MV, metabolic volume; TLG, total lesion glycolysis; SUVmax, maximum standardized uptake value; Si, suspected infection.

The differences in the inguinal DLN-related parameters, including the long diameter, short diameter, maximum cross-sectional area, SUVmax, MV60%, MV70%, MV80%, TLG60%, TLG70%, and TLG80%, and the suspected infection area-related parameters, including SUVmax, MV25%, MV30%, MV35%, MV40%, MV50%, and TLG70%, between the two groups were statistically significant. The efficacy of these parameters to discriminate infection from non-infection were plotted into ROC curves (**Figure 1**). Data of the parameters analyzed with AUCs > 0.8 are summarized in **Table 2**.

**Table 2:** Data of the parameters analyzed with AUC > 0.8.

Category of parameters	Parameters	Infected group /mean ± SD	Non-infected group /mean ± SD	AUC	Sensitivity(%)	Specificity (%)	Cut-off value
Morphological parameters of inguinal DLN	LnSd	10.58 ± 3.62 (mm)	6.38 ± 1.84 (mm)	0.869	86.2	73.7	7.15
	LnLd	21.22 ± 8.28 (mm)	12.65 ± 4.31 (mm)	0.854	84.2	71.9	13.95
	LnCs	242.34 ± 165.04(mm^2^)	84.01 ± 46.15(mm^2^)	0.889	81.2	82.5	117.27
Metabolic parameters of inguinal DLN	LnTLG60%	3.41 ± 2.67	0.84 ± 0.73	0.900	83.2	82.5	1.41
	LnTLG70%	2.13 ± 1.87	0.49 ± 0.41	0.907	85.2	84.2	0.73
	LnTLG80%	1.11 ± 1.02	0.30 ± 0.26	0.870	68.4	93.0	0.55
	LnSUVmax	2.66 ± 1.21	1.08 ± 0.41	0.939	86.8	93.0	1.55
Metabolic parameters of the infection suspected area	SiSUVmax	6.17 ± 2.50	3.65 ± 1.25	0.836	74.0	77.2	4.53

DLN, draining lymph node; Ln, lymph node; Sd, short diameter; Ld, long diameter; Cs, cross-sectional area; TLG, total lesion glycolysis; SUVmax, maximum standardized uptake value; Si, suspected infection.

**Figure 2:**
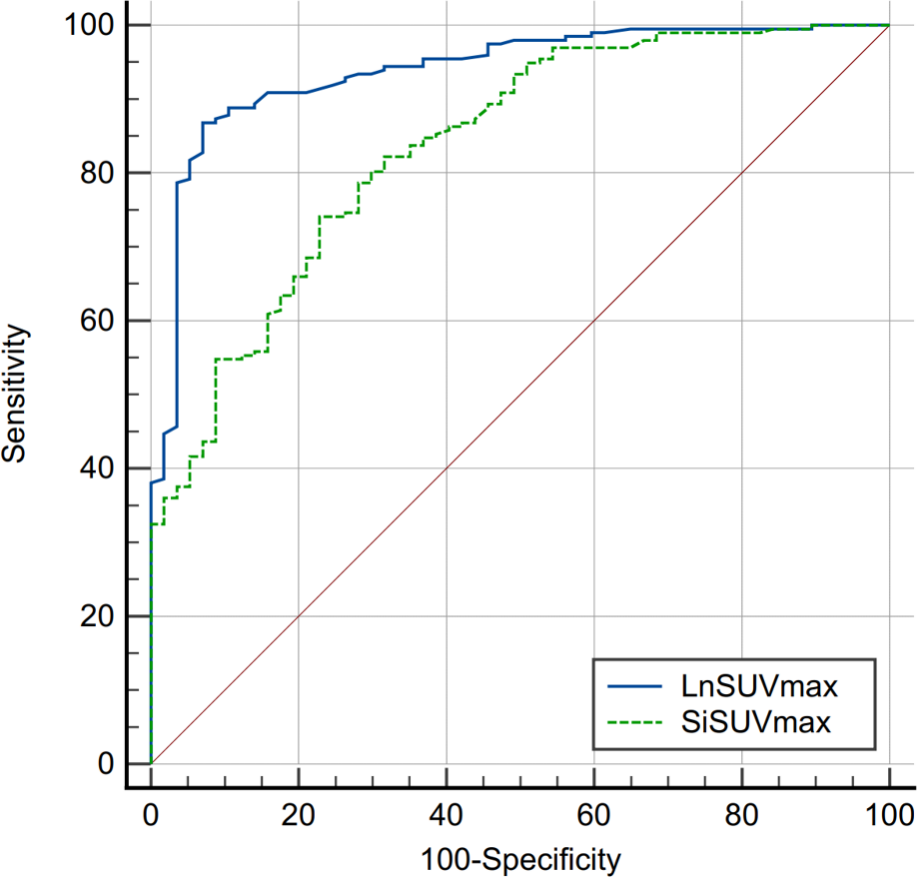
Comparison of receiver operating characteristics (ROC) curves between SUVmax from inguinal draining lymph node (DLN) and suspected infection area (AUC, 0.939 vs 0.836, *P*=0.0006). SUVmax, maximum standardized uptake value; Si, suspected infection.

**Figure 3:**
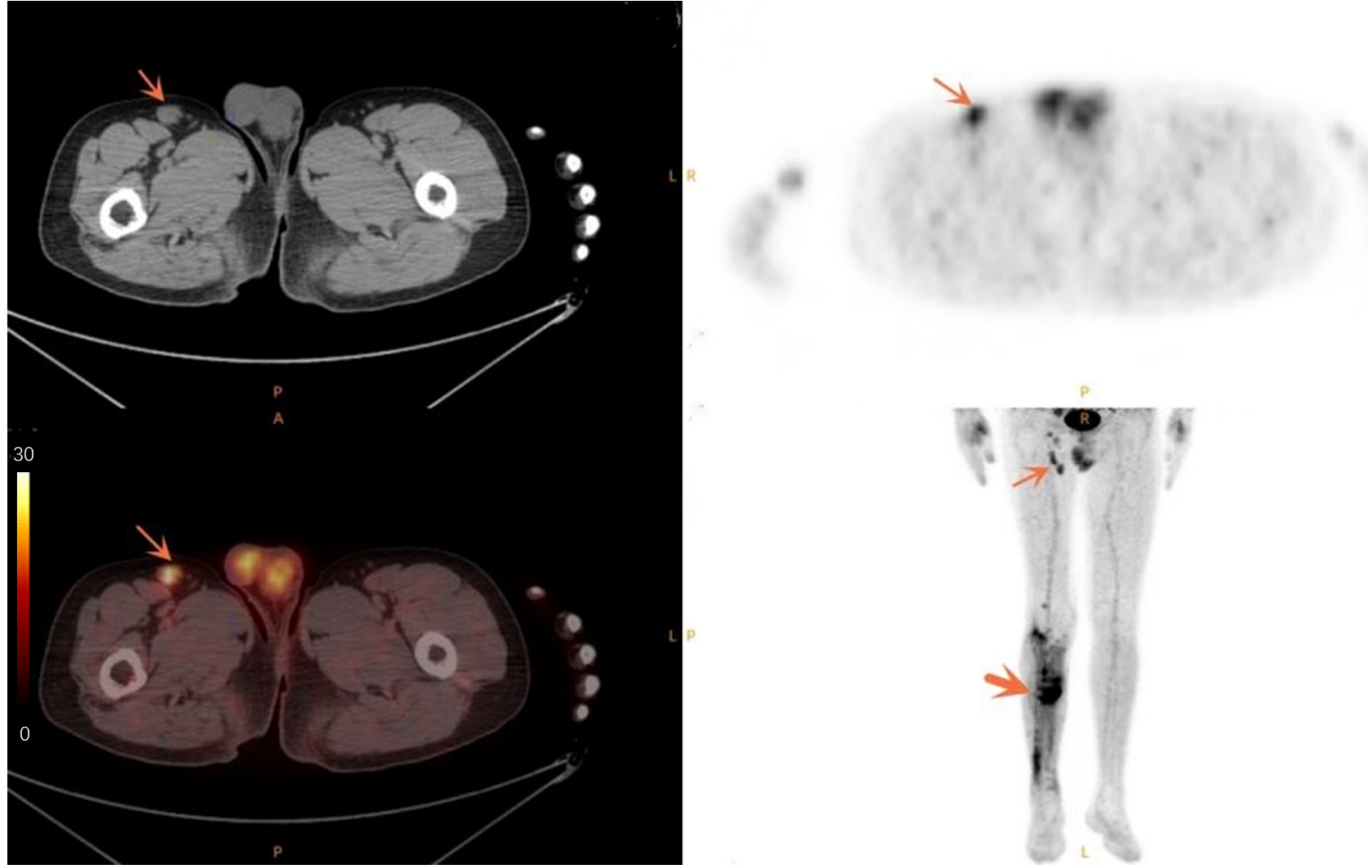
Case of a 50-year-old male patient with infected non-union. The thin arrow points to the swollen lymph node in the right groin with high metabolism. The thick arrow points to the infection area after internal fixation of fracture, and the metabolism of the infected area is increased.

**Figure 4:**
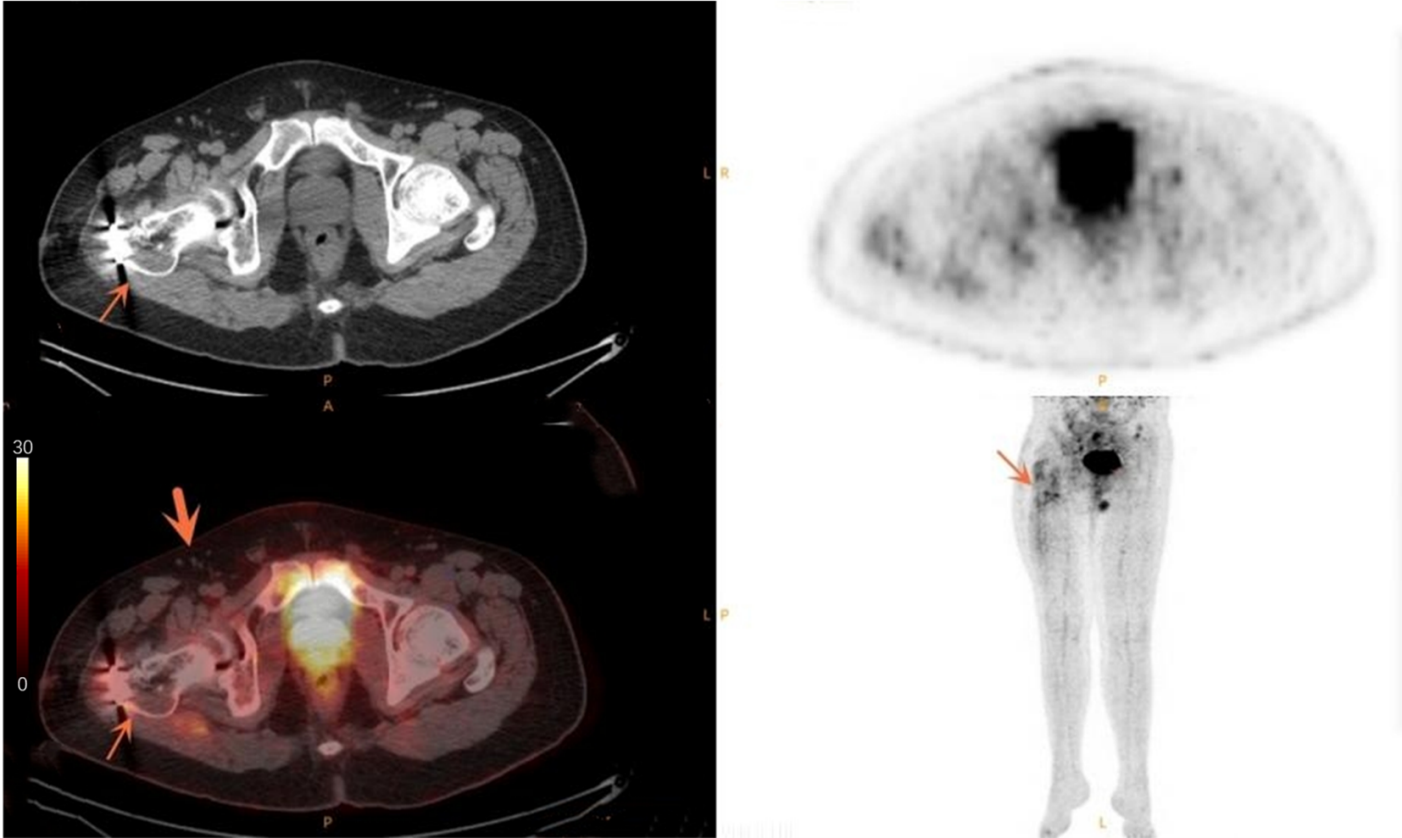
Case of a 33-year-old male patient with simple non-union. The thin arrow points to the increased metabolism after internal fixation of fracture. The thick arrow points to the multiple small lymph nodes in the right groin area without obvious abnormal metabolism.

SUVmax is the easiest parameter obtained in clinical routines. We then compared the AUCs of SUVmax from DLNs and suspected infection areas. The result demonstrated that SUVmax from the inguinal DLNs showed better diagnostic efficiency than SUVmax from suspected infection area (0.939 vs 0.836, *P*=0.0006) (**Figure 2**). Representative cases are provided in **Figures 3**, **4**.

## Discussion

Results of the current study showed that ^18^F- FDG PET/CT could be a promising diagnostic tool for FRI with internal orthopedic implants. Among all the parameters evaluated, SUVmax of the inguinal DLNs showed the best diagnostic accuracy with an AUC of 0.939, sensitivity of 86.8%, and specificity of 93.0%. To the best of our knowledge, the current study is the first one in the literature that investigated the diagnostic value of inguinal DLNs in ^18^F- FDG PET/CT images for FRI in lower extremities.

During the immune response, immune cell activation, infiltration, and proliferation are the basic pathological changes in the DLNs. This process can be etiology and pathogenesis specific, and therefore the immune response in inguinal DLNs, due to chronic infection or sterile inflammation, resulting from fracture-related orthopedic surgery could be different. It is well known that immune cells increase glucose analogue ^18^F-FDG uptake during activation, infiltration, and proliferation because of high energy consumption. So, it is reasonable to believe that the pathognomonic pattern of ^18^F-FDG uptake in FRI could be different from aseptic chronic inflammation not only in suspected infection regions but also in corresponding draining lymph nodes. In this study, we found SUVmax values from suspected infection areas in the lower extremities in the infected group were significantly higher than that in the non-infected group. In addition, values of metabolic parameters, including SUVmax, TLG60%, TLG70%, and TLG80% derived from inguinal DLNs in the infected group, were all significantly higher than those in the non-infected group. Interestingly, the diagnostic efficiency of SUVmax from the inguinal DLN was better than SUVmax from the suspected infection areas in the lower extremities (0.939 vs 0.836, *P*=0.0006).

Vera Wenter et al. (35) investigated the diagnostic value of ^18^F-FDG PET/CT for the detection of chronic osteomyelitis and implant-associated infection and reached a sensitivity of 70% and a specificity of 66% at the cut-off SUVmax value of 3.7. Furthermore, they found that the presence of an orthopedic implant did not reduce the diagnostic yield in patients undergoing PET/CT. Van Vliet et al. (29) used SUVmax to differentiate between aseptic and septic delayed union in the lower extremities. They found a sensitivity, specificity, and diagnostic accuracy of 65%, 77%, and 70%, respectively, at the cut-off SUVmax value of 4.0. Martina Sollini et al. (36) retrospectively tested the diagnostic performances of ^18^F- FDG PET/CT in non-union fractures in 47 patients. The best diagnostic performance was observed when setting the cut-off SUVmax value to 5.92 with an AUC of 0.72. The cut-off SUVmax value identified in their study was higher than in our cohort. Besides differences in patient cohorts and heterogeneous patient populations, the fact that they used microbiological culture results for specimens collected during surgery as the only reference standard to define the final diagnosis could be a significant cause for a higher cut-off value. In real-world clinical practice, the proportion of patients who could not get positive bacterial culture results were not small relatively speaking, as low virulence bacteria such as the coagulasenegative Staphylococcus species may cause culture-negative infection (37). More recently, Lemans et al. (22) reported in a large cohort of patients that the sensitivity and specificity were 80% and 72% at a cut-off SUVmax value of 4.2 for the diagnosis of FRI by using ^18^F-FDG PET/CT. The cut-off value of SUVmax identified in their cohort was very similar to ours. Although ^18^F-FDG PET/CT scans are shown to be promising in the diagnosis of FRI, the fact remains that ^18^F-FDG is a non-specific tracer for infection. Therefore, specific radiopharmaceutical probes targeting microorganisms are warranted in the future.

There are several limitations in this study. Firstly, it is a retrospective study, so there is a potential risk of selection bias. Retrospective studies analyze existing data and cannot control the data collection process, which may result in less accurate and reliable research findings. Secondly, some non-infected patients may not have had sufficient follow-up time, which could lead to false-negative results. Thirdly, some patients may have received antibiotics prior to PET imaging, which could affect the uptake of ^18^F-FDG. Lastly, the number of non-infected patients is significantly smaller than the infected group, leading to data imbalance. This imbalance could result in statistical bias, affecting the reliability of the research results. Future research can improve the reliability of results by increasing the sample size, employing stricter research designs, and controlling for relevant factors.

## Conclusion

In conclusion, our study suggests that semiquantitative analysis (especially SUVmax) of the inguinal draining-lymph node in ^18^F-FDG PET/CT images shows promise as a diagnostic method for detecting FRI with internal fixation after orthopedic surgery in lower extremities. However, further research is necessary to validate and confirm these findings.

## Data availability statement

The original contributions presented in the study are included in the article/supplementary material. Further inquiries can be directed to the corresponding authors.

## Ethics statement

This retrospective study was approved by the ethical review board of the Shanghai Sixth People’s Hospital.

## Author contributions

Research conceived and designed, CS. Data acquisition, YW and ZS. Data analysis interpretation, all authors. Drafted the manuscript, XL, CS, and YW. Revised the manuscript, all authors. Obtained the funding, CS. All authors contributed to the article and approved the submitted version.

## References

[1] Groznik M CM Lusa L Gorenjec NR Ihan A. Increased perioperative C-reactive protein and decreased postoperative albumin is associated with acute posttraumatic osteo- myelitis in patients with high-energy tibial fractures. Injury (2019) 50:827–33. doi: 10.1016/j.injury.2019.02.019 30878258

[2] Buren C Hambüchen M Windolf J Lögters T Windolf CD. Histological score for degrees of severity in an implant-associated infection model in mice. Arch Orthop Trauma Surg (2019) 139(9):1235–44. doi: 10.1007/s00402-019-03188-6 31020411

[3] Ktistakis I Giannoudi M Giannoudis PV. Infection rates after open tibial fractures: are they decreasing? Injury (2014) 45(7):1025–7. doi: 10.1016/j.injury.2014.03.022 24794617

[4] Malhotra AK Goldberg S Graham J Malhotra NR Willis MC Mounasamy V. Open extremity fractures: impact of delay in operative debridement and irrigation. J Trauma Acute Care Surg (2014) 76(5):1201–7. doi: 10.1097/TA.0000000000000205 24747449

[5] TrampuzA Z. Diagnosis and treatment of infections associated with fracture-fixation devices. Injury (2006) 37:S59–66. doi: 10.1016/j.injury.2006.04.010 16651073

[6] Thakore RV Greenberg SE Shi H Foxx AM Francois EL Prablek MA. Surgical site infection in orthopedic trauma: A case-control study evaluating risk factors and cost. J Clin Orthop Trauma (2015) 6(4):220–6. doi: 10.1016/j.jcot.2015.04.004 PMC4600831 26566333

[7] Moriarty TF Kuehl R Coenye T Metsemakers WJ Morgenstern M Schwarz EM. Orthopaedic device-related infection: current and future interventions for improved prevention and treatment. EFORT Open Rev (2016) 1(4):89–99. doi: 10.1302/2058-5241.1.000037 28461934 PMC5367564

[8] Kremers HM Nwojo ME Ransom JE Wood-Wentz CM Melton LJ 3rd Huddleston PM 3rd. Trends in the epidemiology of osteomyelitis: a population-based study, 1969 to 2009. J Bone Joint Surg Am (2015) 97(10):837–45. doi: 10.2106/JBJS.N.01350 PMC4642868 25995495

[9] Metsemakers WJ Morgenstern M Senneville E Borens O Govaert GAM Onsea J. General treatment principles for fracture-related infection: recommendations from an international expert group. Arch Orthop Trauma Surg (2020) 140(8):1013–27. doi: 10.1007/s00402-019-03287-4 PMC7351827 31659475

[10] Metsemakers WJ Morgenstern M McNally MA Moriarty TF McFadyen I Scarborough M. Fracture-related infection: A consensus on definition from an international expert group. Injury (2018) 49(3):505–10. doi: 10.1016/j.injury.2017.08.040 28867644

[11] Hake ME Oh JK Kim JW Ziran B Smith W Hak D. Difficulties and challenges to diagnose and treat post-traumatic long bone osteomyelitis. Eur J Orthop Surg Traumatol (2015) 25(1):1–3. doi: 10.1007/s00590-014-1576-z 25480328

[12] Pineda C Espinosa R Pena A. Radiographic imaging in osteomyelitis: the role of plain radiography, computed tomography, ultrasonography, magnetic resonance imaging, and scintigraphy. Semin Plast Surg (2009) 23(2):80–9. doi: 10.1055/s-0029-1214160 PMC2884903 20567730

[13] Palestro CJ Love C Miller TT. Infection and musculoskeletal conditions: Imaging of musculoskeletal infections. Best Pract Res Clin Rheumatol (2006) 20(6):1197–218. doi: 10.1016/j.berh.2006.08.009 17127204

[14] Lee YJ Sadigh S Mankad K Kapse N Rajeswaran G. The imaging of osteomyelitis. Quant Imaging Med Surg (2016) 6(2):184–98. doi: 10.21037/qims.2016.04.01 PMC4858469 27190771

[15] Buhne KH Bohndorf K. Imaging of posttraumatic osteomyelitis. Semin Musculoskelet Radiol (2004) 8(3):199–204. doi: 10.1055/s-2004-835360 15478023

[16] Kumar R Basu S Torigian D Anand V Zhuang H Alavi A. Role of modern imaging techniques for diagnosis of infection in the era of 18F-fluorodeoxyglucose positron emission tomography. Clin Microbiol Rev (2008) 21(1):209–24. doi: 10.1128/CMR.00025-07 PMC2223836 18202443

[17] Strobel K Stumpe KD. PET/CT in musculoskeletal infection. Semin Musculoskelet Radiol (2007) 11(4):353–64. doi: 10.1055/s-2008-1060337 18324599

[18] Govaert GAM Kuehl R Atkins BL Trampuz A Morgenstern M Obremskey WT. Diagnosing fracture-related infection: current concepts and recommendations. J Orthop Trauma (2020) 34(1):8–17. doi: 10.1097/BOT.0000000000001614 31855973 PMC6903359

[19] Zhang Q Dong J Shen Y Yun C Zhou D Liu F. Comparative diagnostic accuracy of respective nuclear imaging for suspected fracture-related infection: a systematic review and Bayesian network meta-analysis. Arch Orthop Trauma Surg (2021) 141(7):1115–30. doi: 10.1007/s00402-020-03506-3 32514833

[20] Morgenstern M Kuhl R Eckardt H Acklin Y Stanic B Garcia M. Diagnostic challenges and future perspectives in fracture-related infection. Injury (2018) 49 Suppl 1:S83–90. doi: 10.1016/S0020-1383(18)30310-3 29929701

[21] Gholamrezanezhad A Basques K Batouli A Matcuk G Alavi A Jadvar H. Clinical nononcologic applications of PET/CT and PET/MRI in musculoskeletal, orthopedic, and rheumatologic imaging. AJR Am J Roentgenol (2018) 210(6):W245–63. doi: 10.2214/AJR.17.18523 PMC7469956 29787313

[22] Lemans JVC Hobbelink MGG IJpma FFA Plate JDJ van den Kieboom J Bosch P. The diagnostic accuracy of (18)F-FDG PET/CT in diagnosing fracture-related infections. Eur J Nucl Med Mol Imaging (2019) 46(4):999–1008. doi: 10.1007/s00259-018-4218-6 30523391 PMC6450834

[23] Shemesh S Kosashvili Y Groshar D Bernstine H Sidon E Cohen N. The value of 18-FDG PET/CT in the diagnosis and management of implant-related infections of the tibia: a case series. Injury (2015) 46(7):1377–82. doi: 10.1016/j.injury.2015.03.002 25801065

[24] Hartmann A Eid K Dora C Trentz O von Schulthess GK Stumpe KDM. Diagnostic value of 18F-FDG PET/CT in trauma patients with suspected chronic osteomyelitis. Eur J Nucl Med Mol Imaging (2007) 34(5):704–14. doi: 10.1007/s00259-006-0290-4 17136411

[25] Goebel M Rosa F Tatsch K Grillhoesl A Hofmann GO Kirschner MH. [Diagnosis of chronic osteitis of the bones in the extremities. Relative value of F-18 FDG-PET]. Unfallchirurg (2007) 110(10):859–66. doi: 10.1007/s00113-007-1302-y 17805505

[26] Stumpe KD Strobel K. 18F FDG-PET imaging in musculoskeletal infection. Q J Nucl Med Mol Imaging (2006) 50(2):131–42. 16770303

[27] Hsu WK Feeley BT Krenek L Stout DB Chatziioannou AF Lieberman JR. The use of 18F-fluoride and 18F-FDG PET scans to assess fracture healing in a rat femur model. Eur J Nucl Med Mol Imaging (2007) 34(8):1291–301. doi: 10.1007/s00259-006-0280-6 PMC3073515 17334765

[28] Wenter V Albert NL Brendel M Fendler WP Cyran CC Bartenstein P. [(18)F]FDG PET accurately differentiates infected and non-infected non-unions after fracture fixation. Eur J Nucl Med Mol Imaging (2017) 44(3):432–40. doi: 10.1007/s00259-016-3528-9 PMC5591625 27704194

[29] van Vliet KE de Jong VM Termaat MF Schepers T van Eck-Smit BLF Goslings JC. FDG-PET/CT for differentiating between aseptic and septic delayed union in the lower extremity. Arch Orthop Trauma Surg (2018) 138(2):189–94. doi: 10.1007/s00402-017-2806-8 PMC5773632 28956151

[30] Bery AI Shepherd HM Li W Krupnick AS Gelman AE Kreisel D. Role of tertiary lymphoid organs in the regulation of immune responses in the periphery. Cell Mol Life Sci (2022) 79(7):359. doi: 10.1007/s00018-022-04388-x 35689679 PMC9188279

[31] Flandre TD Mansfield KG Espié PJ Rubic-Schneider T Ulrich P. Immunosuppression profile of CFZ533 (Iscalimab), a non-depleting anti-CD40 antibody, and the presence of opportunistic infections in a rhesus monkey toxicology study. Toxicol Pathol (2022) 50(5):712–24. doi: 10.1177/01926233221100168 35730205

[32] Cąkała-Jakimowicz M Puzianowska-Kuznicka M. Towards understanding the lymph node response to skin infection with saprophytic staphylococcus epidermidis. Biomedicines (2022) 10(5):1021. doi: 10.3390/biomedicines10051021 35625758 PMC9138836

[33] Johnson SC Frattolin J Edgar LT Jafarnejad M Moore JE Jr. Lymph node swelling combined with temporary effector T cell retention aids T cell response in a model of adaptive immunity. J R Soc Interface (2021) 18(185):20210464. doi: 10.1098/rsif.2021.0464 34847790 PMC8633806

[34] Pijl JP Nienhuis PH Kwee TC Glaudemans AWJM Slart RHJA Gormsen LC. Limitations and pitfalls of FDG-PET/CT in infection and inflammation. Semin Nucl Med (2021) 51(6):633–45. doi: 10.1053/j.semnuclmed.2021.06.008 34246448

[35] Wenter V Muller JP Albert NL Lehner S Fendler WP Bartenstein P. The diagnostic value of [(18)F]FDG PET for the detection of chronic osteomyelitis and implant-associated infection. Eur J Nucl Med Mol Imaging (2016) 43(4):749–61. doi: 10.1007/s00259-015-3221-4 26547722

[36] Sollini M Trenti N Malagoli E Catalano M Di Mento L Kirienko A. [(18)F]FDG PET/CT in non-union: improving the diagnostic performances by using both PET and CT criteria. Eur J Nucl Med Mol Imaging (2019) 46(8):1605–15. doi: 10.1007/s00259-019-04336-1 31044264

[37] Morgenstern C Cabric S Perka C Trampuz A Renz N. Synovial fluid multiplex PCR is superior to culture for detection of low-virulent pathogens causing periprosthetic joint infection. Diagn Microbiol Infect Dis (2018) 90(2):115–9. doi: 10.1016/j.diagmicrobio.2017.10.016 29191466

